# hs-CRP/HDL Ratio Across Obesity and Type 2 Diabetes Mellitus Phenotypes in Adults: A Cross-Sectional Study

**DOI:** 10.3390/diagnostics16132013

**Published:** 2026-06-27

**Authors:** Crina Cristina Solomon, Nastaca Alina Palade, Felicia Gabriela Gligor, Alina Liliana Pintea, Claudiu Morgovan, Adina Frum, Anca Butuca, Carmen Maximiliana Dobrea, Dragoș Anton Dădârlat, Mariana Cornelia Tilinca

**Affiliations:** 1 Preclinical Department, Faculty of Medicine, “Lucian Blaga” University of Sibiu, 550169 Sibiu, Romania; crinacristina.solomon@ulbsibiu.ro (C.C.S.); claudiu.morgovan@ulbsibiu.ro (C.M.); adina.frum@ulbsibiu.ro (A.F.); anca.butuca@ulbsibiu.ro (A.B.); carmen.dobrea@ulbsibiu.ro (C.M.D.); 2 County Emergency Clinical Hospital Sibiu, 550025 Sibiu, Romania; alina-liliana.pintea@ulbsibiu.ro; 3 Department of Dental Medicine and Nursing, Faculty of Medicine, “Lucian Blaga” University of Sibiu, 550169 Sibiu, Romania; dragos.dadarlat@ulbsibiu.ro; 4 Department of Internal Medicine I, Faculty of Medicine in English, George Emil Palade University of Medicine, Pharmacy, Science, and Technology of Târgu-Mures, 540142 Târgu Mureș, Romania; mariana.tilinca@umfst.ro; 5 Clinical Compartment of Diabetes Mellitus, Nutrition and Metabolic Diseases, Emergency Clinical County Hospital of Targu Mures, 540136 Târgu Mureș, Romania

**Keywords:** obesity, type 2 diabetes, metabolic syndrome, cholesterol, hs-CRP/HDL-C

## Abstract

**Background/Objectives:** Chronic low-grade inflammation and lipid metabolism play an important role in the development of obesity and type 2 diabetes mellitus (T2DM). The ratio of high-sensitivity C-reactive protein to high-density lipoprotein cholesterol (hs-CRP/HDL-C) integrates these pathways and it has been proposed as a novel biomarker of metabolic risk. However, evidence regarding its association with obesity and T2DM remains limited. **Methods:** This retrospective cross-sectional study included data from 413 adults, comprising 251 individuals with T2DM and 162 individuals without T2DM. Obesity status was assessed separately according to BMI categories. The hs-CRP/HDL-C ratio was divided into quartiles, with the lowest quartile used as the reference category. Its associations with obesity and T2DM were evaluated using multivariable logistic regression and restricted cubic spline analysis. Subgroup analyses were conducted to evaluate the consistency of these associations across different population subgroups. **Results:** A significantly positive association was noted between the hs-CRP/HDL-C ratio and the presence of T2DM. Compared with individuals in the lowest quartile, those in the higher quartiles of the hs-CRP/HDL-C ratio had significantly higher odds of T2DM. In the fully adjusted model, each unit increase in the hs-CRP/HDL-C ratio was associated with higher odds of T2DM (OR = 1.59, 95% CI: 1.14–2.20, *p* = 0.006), whereas the association with obesity was attenuated and lost statistical significance after full adjustment. RCS analysis confirmed a significant overall association between the hs-CRP/HDL-C ratio and T2DM risk (*p*-overall = 0.034). Although the hs-CRP/HDL-C ratio was associated with obesity in the crude model (OR = 1.40, 95% CI: 1.05–1.70, *p* = 0.019), the association was not significant after adjustment (OR = 1.18, 95% CI: 0.90–1.56, *p* = 0.229). **Conclusions:** In our study, an elevated hs-CRP/HDL-C ratio was significantly associated with the presence of T2DM and may represent a marker associated with metabolic inflammation, particularly in individuals with glycometabolic disorders.

## 1. Introduction

Worldwide, the prevalence of obesity and diabetes mellitus (DM) is increasing, threatening people’s lives with premature mortality through complications such as cardiovascular disease, renal impairment, and stroke [1]. Moreover, these represent a severe public health problem, increasing healthcare costs, reducing productivity, and causing substantial socioeconomic strain, particularly in low- and middle-income countries where the access to preventive care and treatment may be limited [2]. As lifestyle patterns shift toward urbanization and increased socio-economic growth, all countries are faced with a population with a more stressful and sedentary lifestyle and unhealthy diet habits [3]. The booming population of obesity is predictably contributing to the increase in the prevalence of DM as one of the fastest-growing health challenges of the 21st century [4].

Obesity is increasingly recognized as a chronic inflammatory condition characterized by adipose tissue dysfunction. When excess adipose tissue become dysfunctional, this promotes pro-inflammatory cytokines, including tumor necrosis factor alpha (TNF-α) and interleukine-6 (IL-6), while the release of anti-inflammatory adipokines is reduced [5]. These mediators contribute to systemic low-grade inflammation, oxidative stress, endothelial dysfunction and impaired insulin signaling, ultimately leading to insulin resistance and progressive β cell dysfunction [6]. Consequently, chronic inflammation represents a key mechanistic link between obesity, prediabetes and T2DM [7].

A key feature linking obesity and type 2 diabetes mellitus (T2DM) is represented by the presence of metabolic inflammation, closely intertwined with abnormalities in lipid metabolism by changing the constitution and fraction of the lipid profiles and deteriorating insulin resistance [8]. Diabetic dyslipidemia, a common hallmark in diabetic milieu, appears as a reaction to pro-inflammatory cytokines which alter lipoprotein metabolism, increase triglycerides (TG) and reduce high-density lipoprotein cholesterol (HDL-C) concentrations and functionality [9,10]. Beyond quantitative reductions, HDL-C particles may become dysfunctional under inflammatory conditions, losing part of their anti-atherogenic, anti-inflammatory, antioxidative properties and insulin-sensitizing functions. Therefore, both elevated inflammatory activity and impaired HDL-C function contribute to the development and progression of cardiometabolic disease [11,12]. In addition, metabolic dyslipidemia observed in T2DM is closely associated with chronic low-grade inflammation, reflected by raised levels of high-sensitivity C-reactive protein (hs-CRP) [12]. This chronic inflammation driven by nutrient excess and metabolic imbalance, characterized by the impairment of beta-cells’ functions and enhancement of insulin resistance, will increase acute-phase reactants and other mediators, resulting in the activation of a network of inflammatory signaling pathways [13,14,15]. Particularly, both atherogenic lipid changes and low-grade inflammation have been identified as biologically entangled processes [16]. Previous reports have shown that levels of hs-CRP are significantly elevated in individuals with diabetes and are associated with measures of adiposity [17]. Low-grade systemic inflammation may be used to predict the onset of cardiovascular disease and T2DM, being considered as an effective marker for long term risk assessment [18].

Although each biomarker independently provides clinically relevant information, neither fully shows the complex interaction between inflammation and dyslipidemia that describes obesity and T2DM.

Several surrogate biomarkers have been proposed for the assessment of metabolic and cardiometabolic risk. The triglyceride–glucose (TyG) index is widely used as a marker of insulin resistance, while the TG/HDL-C ratio reflects atherogenic dyslipidemia and impaired insulin sensitivity [19]. More recently, the systemic immune-inflammation index (SII), derived from peripheral blood cell counts, has emerged as an indicator of systemic inflammatory status [20]. Although these indices have demonstrated associations with obesity, T2DM, and cardiovascular disease, each primarily reflects a specific aspect of metabolic dysfunction [21].

In contrast, the hs-CRP/HDL-C ratio has increasingly been recognized as a novel marker that simultaneously integrates these two complementary pathophysiological areas, particularly in individuals with obesity and T2DM [22]. It has been linked to the risks of several chronic conditions, including cardiovascular diseases, metabolic dysfunction-associated fatty liver disease (MAFLD), and hyperuricemia [23]. When evaluated together as a ratio, these biomarkers provide a more integrated view of the inflammatory-lipid imbalance characteristic of metabolic disease than either marker alone [15]. Several studies have reported associations between the hs-CRP/HDL-C ratio and insulin resistance, metabolic syndrome, cardiovascular risk, and adverse metabolic outcomes [24,25]. However, important knowledge gaps remain. Previous investigations have often focused on selected populations, cardiovascular endpoints, or isolated metabolic abnormalities, and the clinical useful of the hs-CRP/HDL-C ratio in individuals with obesity and T2DM remains incompletely characterized. Furthermore, it is unclear whether this clinical marker offers meaningful advantages for cardiometabolic risk stratification compared with traditional inflammatory or lipid biomarkers assessed separately. Additional research is therefore needed to clarify its relationship with metabolic diseases and its potential role as a practical biomarker in clinical settings [25].

Accordingly, this retrospective cross-sectional study aimed to investigate the association between the hs-CRP/HDL-C ratio and the odds of metabolic outcomes, specifically obesity and T2DM, and to evaluate its potential utility as an integrated biomarker of metabolic risk. Moreover, it examined the dose–response relationship and possible interaction effects. We hypothesized that a higher hs-CRP/HDL ratio would be associated with increased odds of obesity and diabetes, and that a nonlinear dose–response association may exist.

## 2. Materials and Methods

### 2.1. Study Population

**Figure 1:**
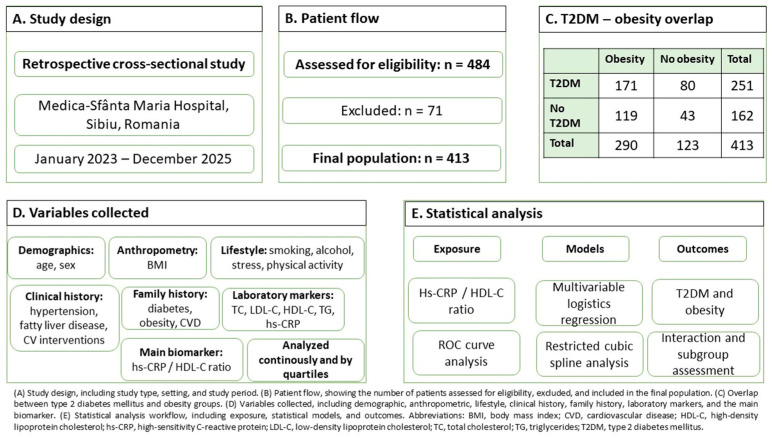
Study population, clinical group overlap, collected variables, and analytical framework.

This retrospective cross-sectional study used clinical data from adult patients evaluated at the Department of Diabetes, Nutrition and Metabolic Diseases of “Medica–Sfânta Maria” Hospital from Sibiu, Romania, between January 2023 and December 2025. Ethical approval was obtained from the institutional ethical committee (No. 64/16 December 2025). In total, 484 adult participants of both sexes, aged 18–90 years, with a confirmed diagnosis of T2DM and/or obesity were screened for eligibility. After excluding 71 cases due to incomplete clinical or laboratory information, 413 participants were included in the final analysis. Of these, 251 participants had T2DM and 162 participants did not have T2DM. Obesity status was assessed separately according to BMI categories and was not mutually exclusive from T2DM. Overall, 290 participants had obesity and 123 participants did not have obesity. Among participants with T2DM, 171 also had obesity, whereas 80 did not have obesity. Among participants without T2DM, 119 had obesity and 43 did not have obesity (Figure 1). This analytical framework allowed for the evaluation of T2DM and obesity as two distinct binary outcomes, while accounting for the overlap between these conditions. In the regression analyses evaluating T2DM as the outcome, individuals without T2DM were used as the reference group. In the regression analyses evaluating obesity as the outcome, individuals without obesity were used as the reference group.

The patient inclusion criteria were age ≥ 18 years, either sex, availability of complete clinical and laboratory data, and the presence of T2DM and/or obesity, including measurements required to calculate the hs-CRP/HDL-C ratio.

The exclusion criteria included incomplete clinical or laboratory data, major comorbidities potentially affecting metabolic or inflammatory outcomes, concomitant endocrine or metabolic disorders other than T2DM or obesity, and acute inflammatory or infectious conditions that could influence hs-CRP levels. To reduce potential bias related to non-metabolic causes of systemic inflammation, patients with documented acute infections, active inflammatory or autoimmune diseases, recent major surgery or trauma, severe hepatic or renal disease, and active malignancy were excluded when such information was available in the medical records. Patients receiving medication with a major potential effect on systemic inflammation, such as systemic corticosteroids, immunosuppressive therapy, or chronic anti-inflammatory treatment, were also excluded when documented.

### 2.2. Definitions of Outcomes/Diagnostic Criteria

According to the American Diabetes Association’s criteria, T2DM was defined based on fasting plasma glucose ≥ 126 mg/dL, 2 h plasma glucose ≥ 200 mg/dL during the oral glucose tolerance test, glycated hemoglobin (HbA1c) ≥ 6.5%, or a prior physician diagnosis. Obesity was defined as body mass index (BMI) ≥ 30 kg/m^2^ according to the World Health Organization’s standards [26].

The primary outcomes used in the regression analyses were the presence of T2DM and the presence of obesity, each coded as a binary variable. For the T2DM outcome, participants with T2DM were compared with participants without T2DM. For the obesity outcome, participants with BMI ≥ 30 kg/m^2^ were compared with those with BMI < 30 kg/m^2^.

Comorbidities, including hypertension, dyslipidemia, hepatic steatosis, chronic venous insufficiency, cancer history, and prior cardiovascular interventions, were extracted from documented clinical diagnoses and assessed to account for the underlying cardiometabolic burden and disease severity, given their potential impact on systemic inflammation and lipid metabolism. Dyslipidemia was defined as elevated total cholesterol (TC) ≥ 200 mg/dL, low-density lipoprotein cholesterol (LDL-C) ≥ 130 mg/dL, triglycerides (TG) ≥ 150 mg/dL, and low HDL-C values, defined as <40 mg/dL in men and <50 mg/dL in women.

The hs-CRP/HDL-C ratio was calculated for each participant by dividing hs-CRP by HDL-C. For statistical analyses, the hs-CRP/HDL-C ratio was analyzed both as a continuous variable and as a categorical variable based on quartiles. For quartile-based analyses, the lowest quartile was used as the reference category. For analyses using the continuous form of the variable, odds ratios were reported per one-unit increase in the hs-CRP/HDL-C ratio.

### 2.3. Data Collection

Clinical data were retrospectively collected from the electronic medical records of “Medica–Sfânta Maria” Clinic from Sibiu. Extracted variables included demographic characteristics, anthropometric parameters, lifestyle-related factors, family medical history, documented clinical comorbidities, and laboratory parameters. Demographic and lifestyle-related variables included age, sex, occupation, educational level, marital status, smoking status, alcohol consumption, physical activity level, and psychological stress. Anthropometric data included height, weight, and body mass index. Family medical history included parental history of T2DM, obesity, or cardiovascular diseases.

Documented clinical comorbidities and relevant medical history were also recorded, including arterial hypertension, prior cardiovascular interventions, metabolic dysfunction-associated steatotic liver disease, cancer history, and chronic venous insufficiency. Information available in the medical records regarding conditions or treatments with potential effects on systemic inflammation was reviewed during data extraction to support the eligibility assessment. Laboratory parameters collected at the time of clinical evaluation included TC, HDL-C, LDL-C, serum TG, and hs-CRP. Anthropometric measurements were obtained according to routine clinical procedures, while laboratory analyses were performed at the hospital’s accredited clinical laboratory using standardized automated assays. Blood samples were collected after an overnight fast, and all measurements were performed in accordance with internal quality control protocols.

### 2.4. Statistical Analysis

Participants were categorized into four groups according to the quartiles of the hs-CRP/HDL-C ratio (Q1–Q4) to explore potential differences in the presence of T2DM and obesity. Continuous variables were described using mean ± SD or median (IQR), as appropriate, while categorical variables were summarized as frequencies and percentages. Participants were stratified according to hs-CRP/HDL-C quartiles, and differences between groups were assessed using appropriate statistical methods based on variable type and distribution. Continuous data were evaluated using either one-way ANOVA or the Kruskal–Wallis test, whereas categorical variables were compared using the chi-square test. The association between the hs-CRP/HDL-C ratio and T2DM was examined through multivariable logistic regression modeling: Model 1 was the crude model, without adjustment. Model 2 was adjusted for age, sex, BMI, marital status, smoking status, alcohol consumption, psychological stress, physical activity level, education level, parental history of T2DM, parental history of obesity, parental history of cardiovascular diseases, hypertension, cardiovascular interventions, MASLD, malignant neoplasm, and chronic venous insufficiency (CVI). Model 3 was further adjusted for lipid parameters, including TC, TG, and LDL-C. A similar modeling strategy was adopted to examine the association between the hs-CRP/HDL-C ratio and obesity.

Covariates were selected based on clinical relevance and their potential role as confounders in the relationship between systemic inflammation, lipid metabolism, T2DM, and obesity. Demographic variables, lifestyle factors, family history, cardiometabolic comorbidities, and lipid-related parameters were included because they may influence both hs-CRP/HDL-C levels and metabolic disease status. To assess potential multicollinearity among the independent variables included in the multivariable models, variance inflation factors were calculated. No relevant multicollinearity was considered present when VIF values were below the conventional threshold of 5. To evaluate the robustness of the findings, sensitivity analyses were performed by comparing progressively adjusted models and by assessing whether the direction and magnitude of the associations remained consistent across models.

To further investigate possible non-linear patterns, restricted cubic spline (RCS) models with four knots were fitted, allowing for a flexible assessment of the association between the hs-CRP/HDL-C ratio and the probability of T2DM and obesity.

Subgroup analyses were conducted as exploratory analyses to assess the consistency of the associations across clinically relevant subgroups. Given the relatively modest sample size, subgroup findings were interpreted cautiously and were considered hypothesis-generating rather than confirmatory.

Associations were quantified using odds ratios (ORs) with their corresponding 95% confidence intervals (CIs). Statistical analyses, including descriptive and logistic regression procedures, were performed in IBM SPSS Statistics version 26.0, whereas R version 4.3.2 was used for RCS modeling and visualization.

## 3. Results

### 3.1. Baseline Characteristics of the Study Population

The final cohort included 413 participants. Of these, 251 participants had T2DM and 162 participants did not have T2DM. Obesity was present in 290 participants and absent in 123 participants. Regarding the overlap between metabolic conditions, 80 participants had isolated T2DM, 119 had isolated obesity, 171 had both T2DM and obesity, and 43 had neither T2DM nor obesity.

The baseline characteristics of the study population across CRP/HDL-C quartiles are presented in Table 1. The final cohort consisted of 249 females (60.2%) and 164 males (39.8%). BMI and nutritional status differed significantly between quartiles, with higher BMI and a greater prevalence of severe obesity observed in the upper quartiles. TG and hs-CRP levels increased progressively across quartiles, whereas HDL-C levels decreased significantly (all *p* < 0.001). In addition, the level of physical activity differed significantly between quartiles (*p* = 0.014). No significant differences were observed for age, sex, lifestyle factors, comorbidities, or other lipid parameters.

### 3.2. Association Between hs-CRP/HDL-C Ratio and T2DM

Logistic regression models were applied to evaluate the association between the hs-CRP/HDL-C ratio and the presence of T2DM (Table 2). Because the hs-CRP/HDL-C ratio had small numerical values, the continuous variable was multiplied by 10 for regression analyses. Therefore, the reported odds ratios for the continuous variable correspond to each one-unit increase in the transformed variable, equivalent to a 0.1-unit increase in the original hs-CRP/HDL-C ratio. In the unadjusted model, higher hs-CRP/HDL-C levels were associated with a significantly higher odds of T2DM (OR = 1.59, 95% CI: 1.25–2.02, *p* < 0.001). This association remained statistically significant after adjustment for demographic and lifestyle variables (OR = 1.67, 95% CI: 1.22–2.29, *p* = 0.002) and persisted in the fully adjusted model (OR = 1.59, 95% CI: 1.14–2.20, *p* = 0.006). The consistency of the effect estimates across the progressively adjusted models supported the robustness of this association. In addition, multicollinearity diagnostics showed no relevant multicollinearity among the covariates included in the multivariable models, with all VIF values being below 1.3. When the hs-CRP/HDL-C ratio was analyzed according to quartiles, participants in the highest quartile had higher odds of T2DM compared with those in the lowest quartile. In the fully adjusted model, participants in Q4 had approximately 2.35-fold higher odds of T2DM than those in Q1 (OR = 2.35, 95% CI: 1.09–5.07, *p* = 0.029). However, the linear trend across quartiles did not reach statistical significance in the fully adjusted model (*p* for trend = 0.079), suggesting that the quartile-based findings should be interpreted cautiously.

### 3.3. Association Between hs-CRP/HDL-C Ratio and Obesity

The hs-CRP/HDL-C ratio was analyzed to evaluate its association with the presence of obesity through logistic regression modeling (Table 3). As in the T2DM analysis, the continuous hs-CRP/HDL-C variable was multiplied by 10 for regression analyses. Therefore, the reported odds ratios for the continuous variable correspond to each one-unit increase in the transformed variable, equivalent to a 0.1-unit increase in the original hs-CRP/HDL-C ratio. In the unadjusted model, a higher hs-CRP/HDL-C ratio was significantly associated with higher odds of obesity (OR = 1.40, 95% CI: 1.05–1.70, *p* = 0.019). However, after adjustment for demographic and lifestyle factors, this association was attenuated and did not remain statistically significant (OR = 1.29, 95% CI: 0.99–1.67, *p* = 0.063). After accounting for all covariates, the association between the hs-CRP/HDL-C ratio and obesity was further attenuated and remained non-significant (OR = 1.18, 95% CI: 0.90–1.56, *p* = 0.229). This attenuation after full adjustment suggests that the association observed in the crude model may be partly explained by confounding cardiometabolic and lipid-related factors rather than reflecting an independent association with obesity. Multicollinearity diagnostics showed no relevant multicollinearity among the covariates included in the multivariable models. Most VIF values were below 1.2, while total cholesterol and LDL-C had higher but acceptable VIF values of approximately 3.

When participants were grouped by quartiles, those in the higher hs-CRP/HDL-C categories showed an initially increased odds of obesity compared with those in the lowest quartile. In the crude model, participants in Q4 had a significantly higher odds of obesity (OR = 2.75, 95% CI: 1.49–5.06, *p* = 0.001). After adjustment for demographic and lifestyle factors, the association remained significant for Q3 (OR = 1.95, 95% CI: 1.01–3.75, *p* = 0.046) and Q4 (OR = 2.42, 95% CI: 1.19–4.94, *p* = 0.015). However, in the fully adjusted model, the associations were attenuated and did not remain statistically significant for any quartile. Trend analysis across quartiles revealed a significant linear association in the crude model (*p* for trend = 0.001) and the partially adjusted model (*p* for trend = 0.013). This association weakened after inclusion of all covariates and did not remain statistically significant in the fully adjusted model (*p* for trend = 0.066), supporting a cautious interpretation of the quartile-based findings for obesity. Overall, these results indicate that the relationship between hs-CRP/HDL-C and obesity was not independent of the full set of adjustment variables.

### 3.4. ROC Curve Analysis of hs-CRP, HDL-C and hs-CRP/HDL-C Ratio

**Figure 2:**
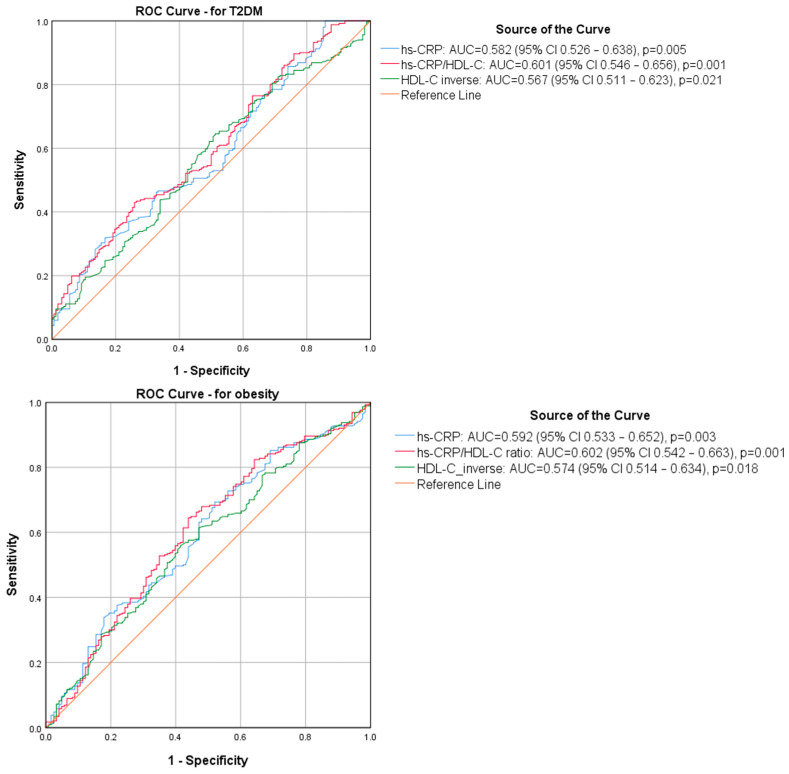
ROC curve analysis for T2DM and obesity.

ROC curve analysis was performed to evaluate whether the hs-CRP/HDL-C ratio provides better discrimination of metabolic disease than hs-CRP or HDL-C assessed separately (Figure 2).

For T2DM, the hs-CRP/HDL-C ratio showed the highest discriminative performance among the analyzed biomarkers, with an AUC of 0.601 (95% CI: 0.546–0.656, *p* = 0.001). This indicates that the combined marker had a slightly better ability to distinguish participants with T2DM from those without T2DM compared with hs-CRP alone (AUC = 0.582, 95% CI: 0.526–0.638, *p* = 0.005) and HDL-C inverse (AUC = 0.567, 95% CI: 0.511–0.623, *p* = 0.021). From a clinical perspective, this suggests that the inflammatory-lipid imbalance captured by the hs-CRP/HDL-C ratio is more closely related to T2DM status than either systemic inflammation or HDL-C reduction considered separately.

For obesity, the hs-CRP/HDL-C ratio also showed the highest AUC among the evaluated biomarkers (AUC = 0.602, 95% CI: 0.542–0.663, *p* = 0.001), followed by hs-CRP alone (AUC = 0.592, 95% CI: 0.533–0.652, *p* = 0.003) and HDL-C inverse (AUC = 0.574, 95% CI: 0.514–0.634, *p* = 0.018). However, the differences between markers were small, indicating that the ratio provides only a limited improvement over isolated biomarkers in distinguishing participants with obesity from those without obesity.

Overall, although the hs-CRP/HDL-C ratio showed the best discriminative performance for both T2DM and obesity, the AUC values were close to 0.60, indicating modest diagnostic discrimination. Therefore, these findings support the hs-CRP/HDL-C ratio as an associated inflammatory-lipid biomarker rather than as a standalone diagnostic tool.

### 3.5. RCS Analysis

**Figure 3:**
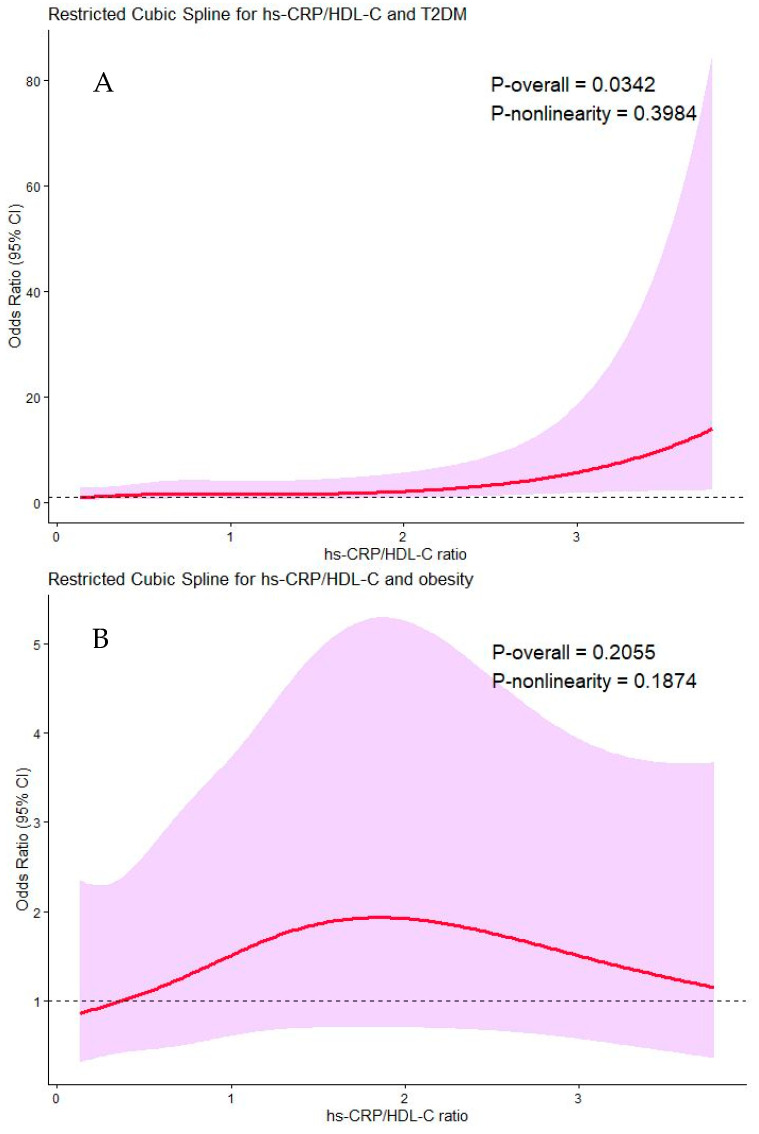
The RCS analysis between the hs-CRP/HDL-C ratio and the association of T2DM and obesity. (**A**) RCS analysis between the hs-CRP/HDL-C ratio and the association of T2DM. (**B**) RCS analysis between the hs-CRP/HDL-C ratio and the association of obesity. The shaded area represents the 95% confidence interval. The black dashed line represents the reference line at odds ratio = 1, indicating no association. The red solid line indicates the estimated association between the hs-CRP/HDL-C ratio and the association of the outcomes, RCS, restricted cubic spline; CI, confidence interval, T2DM, type 2 diabetes mellitus, hs-CRP: high-sensitivity C reactive protein, HDL-C: high-density lipoprotein cholesterol.

To investigate possible non-linear relationships, restricted cubic spline (RCS) models were fitted for the hs-CRP/HDL-C ratio in relation to the presence of T2DM and obesity. The analysis revealed a significant overall association between the hs-CRP/HDL-C ratio and the odds of T2DM (*p*-overall = 0.0342). However, the test for non-linearity was not statistically significant (*p*-nonlinearity = 0.3984), suggesting that the relationship between the hs-CRP/HDL-C ratio and T2DM followed a predominantly linear increasing pattern. As the hs-CRP/HDL-C ratio increased, the estimated odds of T2DM progressively increased, particularly at higher ratio values (Figure 3A). These findings support an association between higher hs-CRP/HDL-C values and T2DM, although they should not be interpreted as evidence of risk prediction due to the cross-sectional design of the study. In contrast, the RCS analysis for obesity did not show a statistically significant association between the hs-CRP/HDL-C ratio and the odds of obesity (*p*-overall = 0.2055). The test for non-linearity was also not significant (*p*-nonlinearity = 0.1874), indicating that no clear linear or nonlinear relationship was observed between the hs-CRP/HDL-C ratio and obesity in the fully adjusted model (Figure 3B). This finding is consistent with the logistic regression results, in which the association with obesity was attenuated after full adjustment.

### 3.6. Subgroup Analyses

Exploratory subgroup and interaction analyses were conducted to evaluate whether the relationship between the hs-CRP/HDL-C ratio and the presence of T2DM and obesity differed across clinically relevant strata, including sex, age, BMI, smoking status, alcohol intake, psychological stress, hypertension, fatty liver disease, and triglyceride levels.

**Figure 4:**
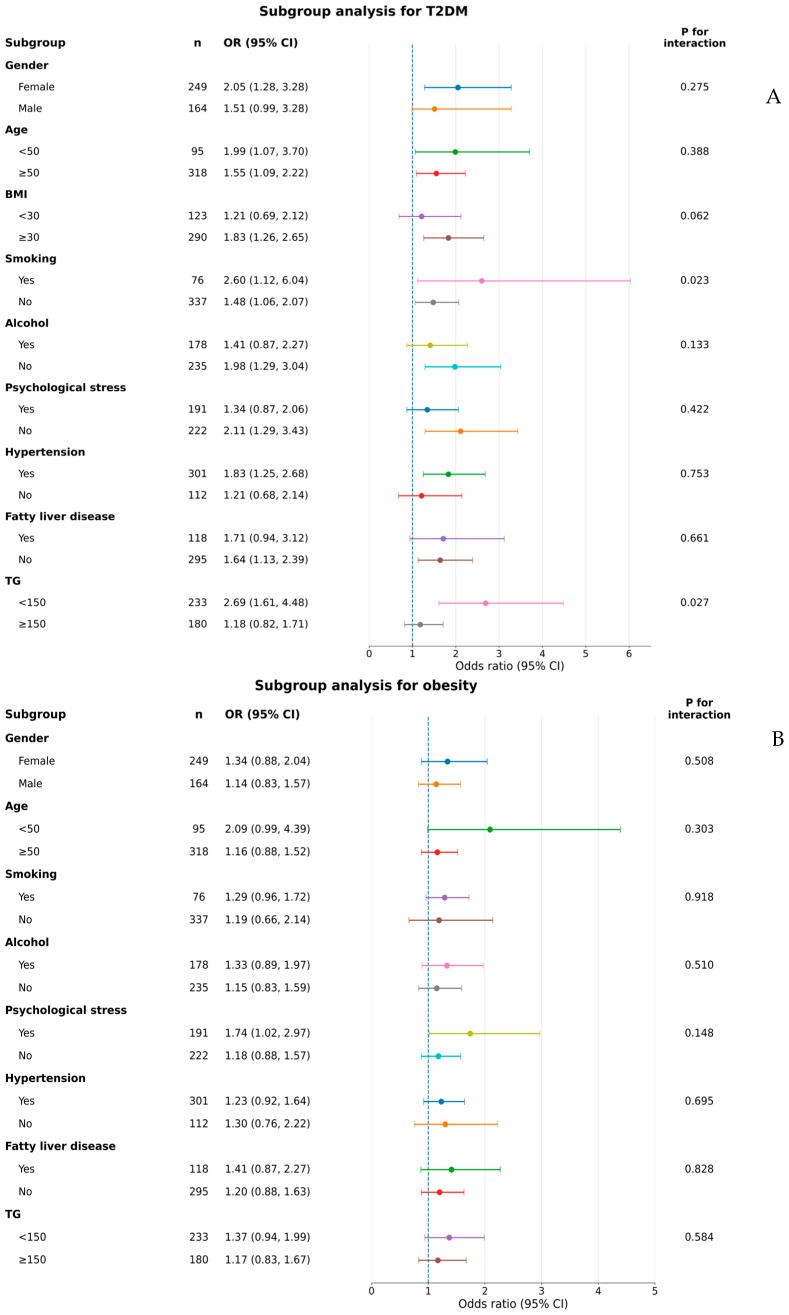
Subgroup analysis for the association between the hs-CRP/HDL-C ratio and the association of T2DM and obesity. (**A**) Subgroup analysis for the association between the hs-CRP/HDL-C ratio and the association of T2DM. (**B**) Subgroup analysis for the association between the hs-CRP/HDL-C ratio and the association of obesity. Abbreviations: BMI, body mass index; TG, triglycerides; OR, odds ratio; CI, confidence interval; hs-CRP, high-sensitivity C-reactive protein; HDL-C, high-density lipoprotein cholesterol.

A positive relationship between the hs-CRP/HDL-C ratio and the odds of T2DM was observed in the majority of the examined subgroups (Figure 4A). Stronger associations were noted among females, individuals with BMI ≥ 30 kg/m^2^, smokers, and participants with triglyceride levels < 150 mg/dL. Interaction tests indicated significant heterogeneity for smoking status and TG levels (both *p* for interaction < 0.05), suggesting that these variables may modify the strength of the association between the hs-CRP/HDL-C ratio and T2DM. However, given the reduced sample size within stratified analyses, these subgroup findings should be interpreted cautiously and considered hypothesis-generating rather than confirmatory.

For obesity, the hs-CRP/HDL-C ratio showed a generally positive but weaker association across subgroups (Figure 4B). Interaction analyses did not identify significant variation across the investigated factors (all *p* for interaction > 0.05), indicating that the association between the hs-CRP/HDL-C ratio and obesity was broadly consistent across the analyzed subgroups. Nevertheless, because the main fully adjusted association with obesity was not statistically significant, these subgroup findings should also be interpreted cautiously and should not be considered evidence of an independent association.

## 4. Discussion

This retrospective cross-sectional study of 413 participants found an association between the hs-CRP/HDL-C ratio and the presence of T2DM and obesity. Higher levels of the hs-CRP/HDL-C ratio were significantly associated with higher odds of T2DM, whereas the association with obesity was attenuated after adjustments for potential confounding variables. Importantly, the independent association between the hs-CRP/HDL-C ratio and T2DM appeared stronger and more consistent than the association observed with obesity. These results suggest that the hs-CRP/HDL-C ratio may represent a useful marker associated with the interplay between systemic inflammation and lipid metabolism disturbances in metabolic disorders.

In the present analysis, individuals in the higher quartiles of the hs-CRP/HDL-C ratio presented altered metabolic profiles, including higher BMI, elevated TG levels, and lower HDL-C concentrations. These findings are consistent with previous evidence showing that obesity and T2DM are commonly associated with chronic low-grade inflammation and lipid metabolism abnormalities. Chronic inflammation and excessive adipose tissue have also been linked to insulin resistance and adipocyte dysfunction [25]. Furthermore, logistic regression analysis showed that the hs-CRP/HDL-C ratio remained independently associated with T2DM after adjusting for demographic, lifestyle, and metabolic factors. In contrast, the association with obesity did not remain statistically significant after full adjustment, suggesting that the crude relationship may be largely explained by overlapping cardiometabolic and lipid-related factors [27]. The RCS analysis showed a progressive increase in the estimated odds of T2DM with increasing hs-CRP/HDL-C values, supporting a predominantly linear association. However, given the cross-sectional design, these findings should not be interpreted as evidence of causality or association prediction.

Our findings are consistent with previous epidemiological studies evaluating inflammatory-lipid ratios in cardiometabolic diseases. For instance, Xue K. et al. reported that higher hs-CRP/HDL-C ratios were associated with increased odds of prediabetes and T2DM in a population-based analysis using NHANES data [28]. Similarly, other studies reported that elevated hs-CRP/HDL-C ratios were associated with adverse cardiometabolic profiles and a higher prevalence of metabolic syndrome [29]. Han F. et al. also suggested that the hs-CRP/HDL-C ratio may be more strongly associated with cardiometabolic risk than hs-CRP or HDL-C alone [30]. These findings support the idea that combined inflammatory and lipid biomarkers may better reflect the complex pathophysiology of metabolic disorders than isolated biomarkers [28,31]. The novelty of the present study lies in the simultaneous evaluation of the hs-CRP/HDL-C ratio in relation to both T2DM and obesity, while accounting for the overlap between these conditions and showing that the independent association is more robust for T2DM than for obesity. Several biological mechanisms may explain the association between the hs-CRP/HDL-C ratio and metabolic disorders. Chronic low-grade inflammation is frequently observed in insulin resistance and T2DM [30,32]. Pro-inflammatory cytokines may impair insulin signaling, reduce glucose uptake, and contribute to β-cell dysfunction [33]. hs-CRP is an acute-phase protein produced by the liver in response to inflammatory cytokines, particularly interleukin-6, and is widely used as a marker of systemic inflammation [34]. Although hs-CRP is mainly considered an inflammatory marker, previous evidence suggests that CRP may also be involved in endothelial dysfunction, vascular inflammation, and metabolic dysregulation. HDL-C has a protective role in metabolic and cardiovascular health. HDL particles are involved in reverse cholesterol transport and have anti-inflammatory, antioxidant, and endothelial-protective properties [35]. In inflammatory states, HDL particles may become dysfunctional and lose part of their anti-inflammatory and antioxidant effects [32]. This is particularly relevant in metabolic disease, where low-grade inflammation and lipid abnormalities often coexist. Therefore, the hs-CRP/HDL-C ratio may reflect the balance between pro-inflammatory activity and reduced HDL-mediated protection [28,35]. A higher ratio may indicate a metabolic environment associated with insulin resistance and metabolic dysfunction [28,30].

Adipose tissue also plays an important role in systemic inflammation. Adipocytes and infiltrating immune cells secrete pro-inflammatory cytokines, including tumor necrosis factor-α and interleukin-6, which stimulate hepatic CRP synthesis and contribute to persistent low-grade inflammation [36,37]. However, in the present study, the association between the hs-CRP/HDL-C ratio and obesity was no longer significant after full adjustment. This suggests that the hs-CRP/HDL-C ratio may be more closely related to T2DM-associated metabolic inflammation than to obesity alone.

The hs-CRP/HDL-C ratio has several potential advantages as a clinical marker. Both hs-CRP and HDL-C are routinely measured in clinical practice, making the ratio easy to calculate without additional costs. Moreover, the ratio combines inflammatory and lipid-related information, which may be relevant in cardiometabolic disease assessment [28,31]. However, the clinical applicability of this ratio should be interpreted cautiously. The present study did not assess predictive performance, diagnostic accuracy, cut-off values, or longitudinal outcomes. Therefore, the hs-CRP/HDL-C ratio should be considered an associated marker of metabolic inflammation rather than an established clinical prediction tool. Further prospective studies are needed to determine whether this ratio can improve risk stratification or guide preventive and therapeutic strategies.

This study has several strengths. First, it provides a comprehensive analysis of the relationship between the hs-CRP/HDL-C ratio and metabolic outcomes using logistic regression and RCS analysis. Second, the inclusion of multiple potential confounders enhances the analytical rigor of the findings. Third, the study specifically considered the overlap between T2DM and obesity, allowing for a more nuanced interpretation of the independent associations with each condition.

However, several limitations should be acknowledged. First, the cross-sectional design limits the ability to infer causal relationships or temporal direction between the hs-CRP/HDL-C ratio and metabolic outcomes. Second, although several covariates were included in the multivariable models, residual confounding cannot be excluded, particularly from unmeasured dietary, pharmacological, genetic, or inflammatory factors. In particular, complete and reliable information regarding medication use, including statins, metformin, insulin, GLP-1 receptor agonists, and anti-inflammatory drugs, was not available for all participants. Since these treatments may influence hs-CRP and HDL-C levels, medication-related adjustment could not be performed and this may have affected the observed associations. Third, the study was conducted in a single center, which may limit the generalizability of the findings. Fourth, the exclusion of patients with incomplete data or conditions that could influence hs-CRP may have introduced selection bias. Fifth, subgroup analyses were limited by modest sample sizes after stratification, which may have resulted in unstable estimates; therefore, these findings should be considered exploratory and hypothesis-generating. Finally, the absence of longitudinal follow-up prevents an evaluation of whether the hs-CRP/HDL-C ratio predicts the future development of T2DM, obesity progression, or cardiometabolic events.

In conclusion, the hs-CRP/HDL-C ratio was independently associated with T2DM, while its association with obesity was attenuated and no longer significant after full adjustment. These findings suggest that the hs-CRP/HDL-C ratio may better reflect inflammatory-lipid disturbances related to T2DM than obesity alone. Nevertheless, because of the retrospective cross-sectional design, the results should be interpreted as associations and not as evidence of causality or predictive clinical utility.

## 5. Conclusions

In conclusion, the present study showed that higher hs-CRP/HDL-C ratio levels are significantly associated with higher odds of T2DM, particularly after adjustment for demographic, lifestyle, and metabolic factors. In contrast, the association between the hs-CRP/HDL-C ratio and obesity was attenuated and lost statistical significance after full adjustment, suggesting that this relationship may be largely explained by overlapping cardiometabolic and lipid-related factors. These findings support the role of the hs-CRP/HDL-C ratio as a potential biomarker reflecting the interaction between systemic inflammation and lipid metabolism disturbances, especially in relation to T2DM. Given the retrospective cross-sectional design, the hs-CRP/HDL-C ratio should be interpreted as an associated marker rather than as a predictive or risk-stratification tool. Since both hs-CRP and HDL-C are routinely measured in clinical practice, this ratio may represent a simple and accessible marker for further evaluation in cardiometabolic research. Further prospective and longitudinal studies are required to confirm these findings and to determine whether the hs-CRP/HDL-C ratio has true predictive value for cardiometabolic disease progression and clinical outcomes.

## Figures and Tables

**Table 1:** Baseline characteristics of the study population according to hs-CRP/HDL-C quartiles.

Characteristic	Overall (100%)	Q1 (0.009–0.392)	Q2 (0.397–0.818)	Q3 (0.823–1.539)	Q4 (1.556–7.640)	*p*-Value
No. of Patients	413	*n* = 103	*n* = 103	*n* = 104	*n* = 103	
Age, Mean ± SD	59.51 ± 13.35	59.34 ± 13.94	61.79 ± 12.05	58.06 ± 14.28	58.86 ± 12.91	0.214
Sex, *n* (%)						0.163
Male	164 (39.7)	39 (37.9)	40 (38.8)	35 (33.7)	50 (48.5)	
Female	249 (60.3)	64 (62.1)	63 (61.2)	69 (66.3)	53 (51.5)	
BMI, Median (IQR)	32.35 (28.91, 36.68)	30.36 (27.76, 33.96)	32.19 (29.04, 35.24)	32.96 (29.39, 37.61)	34.29 (30.25, 39.64)	<0.001
Nutritional status, *n* (%)						0.004
Underweight	2 (0.5)	2 (1.9)	0 (0.0)	0 (0.0)	0 (0.0)	
Normal weight	28 (6.8)	12 (11.7)	8 (7.8)	5 (4.8)	3 (2.9)	
Overweight	109 (26.4)	36 (35.0)	27 (26.2)	26 (25.0)	20 (19.4)	
Obese I	140 (33.9)	32 (31.0)	39 (37.9)	34 (32.7)	35 (34.0)	
Obese II	76 (18.4)	14 (13.6)	19 (18.4)	23 (22.1)	20 (19.4)	
Obese III	58 (14.0)	7 (6.8)	10 (9.7)	16 (15.4)	25 (24.3)	
Marital status, *n* (%)						0.955
Single	31 (7.5)	8 (7.8)	7 (6.8)	8 (7.7)	8 (7.8)	
Married	264 (63.9)	66 (64.1)	64 (62.1)	67 (64.4)	67 (65.0)	
Divorced	41 (9.9)	10 (9.7)	7 (6.8)	12 (11.5)	12 (11.7)	
Widowed	77 (18.7)	19 (18.4)	25 (24.3)	17 (16.4)	16 (15.5)	
Current smoking, *n* (%)						0.357
Yes	76 (18.4)	18 (17.5)	14 (13.6)	24 (23.1)	20 (19.4)	
No	337 (81.6)	85 (82.5)	89 (86.4)	80 (76.9)	83 (80.6)	
Ex-smoking, *n* (%)						0.864
Yes	105 (25.4)	23 (22.3)	27 (26.2)	27 (26.0)	28 (27.2)	
No	308 (74.6)	80 (77.7)	76 (73.8)	77 (74.0)	75 (72.8)	
Never smoking, *n* (%)						0.389
Yes	1 (0.2)	0 (0.0)	0 (0.0)	0 (0.0)	1 (1.0)	
No	412 (99.8)	103 (100)	103 (100)	104 (0.0)	102 (99.0)	
Drinking, *n* (%)						0.760
Yes	178 (43.1)	49 (47.6)	42 (40.8)	44 (42.3)	43 (41.7)	
No	235 (56.9)	54 (52.4)	61 (59.2)	60 (57.7)	60 (58.3)	
Psychological stress, *n* (%)						0.291
Yes	191 (46.2)	43 (41.7)	50 (48.5)	55 (52.9)	43 (41.7)	
No	222 (53.8)	60 (58.3)	53 (51.5)	49 (47.1)	60 (58.3)	
Level of physical activity, *n* (%)						0.014
Sedentary	124 (30.0)	39 (37.9)	32 (31.1)	28 (26.9)	25 (24.3)	
Light activity	24 (5.8)	0 (0.0)	6 (5.8)	6 (5.8)	12 (11.7)	
Moderate activity	265 (64.2)	64 (62.1)	65 (63.1)	70 (67.3)	66 (64.1)	
Education, *n* (%)						0.262
Primary school	43 (10.4)	9 (8.7)	6 (5.8)	12 (11.5)	16 (15.5)	
Secondary school	242 (58.6)	64 (62.1)	61 (59.2)	64 (61.5)	53 (51.5)	
Higher school	128 (31.0)	30 (29.1)	36 (35.0)	28 (26.9)	34 (33.0)	
Parents with T2DM, *n* (%)						0.834
Yes	151 (36.6)	34 (33.0)	40 (38.8)	38 (36.5)	39 (37.9)	
No	262 (63.4)	69 (67.0)	63 (61.2)	66 (63.5)	64 (62.1)	
Parents with obesity, *n* (%)						0.120
Yes	119 (28.8)	21 (20.4)	35 (34.0)	29 (27.9)	34 (33.0)	
No	294 (71.2)	82 (79.6)	68 (66.0)	75 (72.1)	69 (67.0)	
Parents with CVDs, *n* (%)						0.325
Yes	224 (54.2)	60 (58.3)	49 (47.6)	61 (58.7)	54 (52.4)	
No	189 (45.8)	43 (41.7)	54 (52.4)	43 (41.3)	49 (47.6)	
Hypertension, *n* (%)						0.740
Yes	301 (72.9)	72 (69.9)	75 (72.8)	75 (72.1)	79 (76.7)	
No	112 (27.1)	31 (30.1)	28 (27.2)	29 (27.9)	24 (23.3)	
CV, *n* (%)						0.439
Yes	44 (10.7)	10 (9.7)	11 (10.7)	8 (7.7)	15 (14.6)	
No	369 (89.3)	93 (90.3)	92 (89.3)	96 (92.3)	88 (85.4)	
Fatty liver disease, *n* (%)						0.325
Yes	118 (28.6)	25 (24.3)	26 (25.2)	36 (34.6)	31 (30.1)	
No	295 (71.4)	78 (75.7)	77 (74.8)	68 (65.4)	72 (69.9)	
Malignant neoplasm, *n* (%)						0.403
Yes	21 (5.1)	6 (5.8)	2 (1.9)	7 (6.7)	6 (5.8)	
No	392 (94.9)	97 (94.2)	101 (98.1)	97 (93.3)	97 (94.2)	
CVI, *n* (%)						0.912
Yes	43 (10.4)	11 (10.7)	12 (11.7)	9 (8.7)	11 (10.7)	
No	370 (89.6)	92 (89.3)	91 (88.3)	95 (91.3)	92 (89.3)	
TC (mg/dL), Median (IQR)	203 (171, 233)	197 (164.0, 230.3)	203 (175.0, 228.5)	216 (173.5, 246.0)	200 (166.7, 228.5)	0.191
TG (mg/dL), Median (IQR)	141 (103.0, 201.8)	113 (82.5, 157.9)	137 (111.5, 202.5)	147 (103.7, 208.0)	162 (119.5, 255.0)	<0.001
HDL-C (mg/dL), Median (IQR)	48.3 (40.0, 56.9)	52 (42.6, 58.0)	49 (42.0, 68.4)	49 (41.2, 57.0)	42 (37.0, 50.3)	<0.001
LDL-C (mg/dL), Median (IQR)	127 (94.0, 156.0)	125 (91.0, 153.2)	122 (92.0, 152.5)	135 (98.0, 165.2)	126 (92.5, 147.6)	0.406
hs-CRP (mg/L), Median (IQR)	3.98 (1.89, 7.12)	1.29 (1.03, 1.63)	2.89 (2.36, 3.56)	5.80 (4.30, 6.80)	9.78 (7.96, 12.3)	<0.001
hs-CRP/HDL-C, Median (IQR)	0.82 (0.39, 1.54)	0.26 (0.19, 0.32)	0.59 (0.49, 0.70)	1.14 (0.99, 1.33)	2.31 (1.84, 2.93)	<0.001

Note: T2DM: type 2 diabetes mellitus, BMI: body mass index, hs-CRP: high-sensitivity C reactive protein, CVI: Chronic venous insufficiency, HDL-C: high-density lipoprotein cholesterol, LDL-C: low-density lipoprotein cholesterol, TC: total cholesterol, TG: triglycerides.

**Table 2:** The associations between hs-CRP/HDL-C ratio and T2DM.

Exposure Variable	Model 1		Model 2		Model 3	
	OR (95% CI)	*p*-Value	OR (95% CI)	*p*-Value	OR (95% CI)	*p*-Value
(hs-CRP/HDL-C) × 10	1.59 (1.25, 2.02)	<0.001	1.67 (1.22, 2.29)	0.002	1.59 (1.14, 2.20)	0.006
Quartile						
Q1	1 (Ref)		1 (Ref)		1 (Ref)	
Q2	1.749 (1.001, 3.056)	0.049	1.81 (0.90, 3.63)	0.097	1.76 (0.86, 3.61)	0.123
Q3	1.286 (0.744, 2.223)	0.368	1.31 (0.66, 2.58)	0.437	1.23 (0.61, 2.49)	0.559
Q4	2.503 (1.405, 4.459)	0.002	2.66 (1.27, 5.59)	0.010	2.35 (1.09, 5.07)	0.029
*p* for trend	0.013		0.031		0.079	

Note: Model 1: Non-adjusted; Model 2: Adjusted for age, sex, BMI, marital status, current smoking, ex-smoking, never smoking, drinking, psychological stress, level of physical activity, education, parents with T T2DM, parents with obesity, parents with CVDs, hypertension, cardiovascular interventions, fatty liver disease, malignant neoplasm, chronic venous insufficiency (CVI); Model 3: Included additional adjustments for TC, TG, LDL-C. The continuous hs-CRP/HDL-C variable was multiplied by 10 for regression analyses; therefore, odds ratios correspond to each one-unit increase in the transformed variable, equivalent to a 0.1-unit increase in the original hs-CRP/HDL-C ratio. Abbreviations: OR: odds ratio; CI: confidence interval; T2DM: type 2 diabetes mellitus, BMI: body mass index, hs-CRP: high-sensitivity C reactive protein, HDL-C: high-density lipoprotein cholesterol.

**Table 3:** The associations between hs-CRP/HDL-C ratio and obesity.

Exposure Variable	Model 1		Model 2		Model 3	
	OR (95% CI)	*p*-Value	OR (95% CI)	*p*-Value	OR (95% CI)	*p*-Value
(hs-CRP/HDL-C) × 10	1.40 (1.05, 1.70)	0.019	1.29 (0.99, 1.67)	0.063	1.18 (0.90, 1.56)	0.229
Quartile						
Q1	1 (Ref)		1 (Ref)		1 (Ref)	
Q2	1.82 (1.02, 3.23)	0.043	1.85 (0.95, 3.61)	0.072	1.69 (0.85, 3.34)	0.132
Q3	2.13 (1.18, 3.83)	0.012	1.95 (1.01, 3.75)	0.046	1.78 (0.91, 3.47)	0.093
Q4	2.75 (1.49, 5.06)	0.001	2.42 (1.19, 4.94)	0.015	1.95 (0.93, 4.10)	0.077
*p* for trend	0.001		0.013		0.066	

Note: Model 1: Non-adjusted; Model 2: Adjusted for age, sex, marital status, current smoking, ex-smoking, never smoking, drinking, psychological stress, level of physical activity, education, parents with T2DM, parents with obesity, parents with CVDs, hypertension, cardiovascular interventions, fatty liver disease, malignant neoplasm, chronic venous insufficiency (CVI); Model 3: Included additional adjustments for TC, TG, LDL-C. The continuous hs-CRP/HDL-C variable was multiplied by 10 for regression analyses; therefore, odds ratios correspond to each one-unit increase in the transformed variable, equivalent to a 0.1-unit increase in the original hs-CRP/HDL-C ratio. Abbreviations: OR: odds ratio; CI: confidence interval; T2DM: type 2 diabetes mellitus, hs-CRP: high-sensitivity C reactive protein, HDL-C: high-density lipoprotein cholesterol.

## Data Availability

The original contributions presented in this study are included in the article. Further inquiries can be directed to the corresponding authors.

## References

[1] Ruze R., Liu T., Zou X., Song J., Chen Y., Xu R., Yin X., Xu Q. (2023). Obesity and Type 2 Diabetes Mellitus: Connections in Epidemiology, Pathogenesis, and Treatments. Front. Endocrinol..

[2] Kumanyika S., Dietz W.H. (2020). Solving Population-Wide Obesity—Progress and Future Prospects. N. Engl. J. Med..

[3] Hruby A., Hu F.B. (2015). The Epidemiology of Obesity: A Big Picture. Pharmacoeconomics.

[4] Sun H., Saeedi P., Karuranga S., Pinkepank M., Ogurtsova K., Duncan B.B., Stein C., Basit A., Chan J.C.N., Mbanya J.C. (2022). IDF Diabetes Atlas: Global, Regional and Country-Level Diabetes Prevalence Estimates for 2021 and Projections for 2045. Diabetes Res. Clin. Pract..

[5] Hajer G.R., van Haeften T.W., Visseren F.L.J. (2008). Adipose Tissue Dysfunction in Obesity, Diabetes, and Vascular Diseases. Eur. Heart J..

[6] Yudkin J.S., Stehouwer C.D.A., Emeis J.J., Coppack S.W. (1999). C-Reactive Protein in Healthy Subjects: Associations with Obesity, Insulin Resistance, and Endothelial Dysfunction. Arterioscler. Thromb. Vasc. Biol..

[7] Maachi M., Piéroni L., Bruckert E., Jardel C., Fellahi S., Hainque B., Capeau J., Bastard J.P. (2004). Systemic Low-Grade Inflammation Is Related to Both Circulating and Adipose Tissue TNFα, Leptin and IL-6 Levels in Obese Women. Int. J. Obes..

[8] Petersmann A., Müller-Wieland D., Müller U.A., Landgraf R., Nauck M., Freckmann G., Heinemann L., Schleicher E. (2019). Definition, Classification and Diagnosis of Diabetes Mellitus. Exp. Clin. Endocrinol. Diabetes.

[9] Vasudevan A.R., Garber A.J. (2006). Diabetic Dyslipidemia and the Heart. Heart Fail. Clin..

[10] Biondi-Zoccai G.G.L., Abbate A., Liuzzo G., Biasucci L.M. (2003). Atherothrombosis, Inflammation, and Diabetes. J. Am. Coll. Cardiol..

[11] Ashen M.D., Blumenthal R.S. (2005). Low HDL Cholesterol Levels. N. Engl. J. Med..

[12] Wellen K.E., Hotamisligil G.S. (2005). Inflammation, Stress, and Diabetes. J. Clin. Investig..

[13] Hansen S.E.J., Madsen C.M., Varbo A., Nordestgaard B.G. (2019). Low-Grade Inflammation in the Association between Mild-to-Moderate Hypertriglyceridemia and Risk of Acute Pancreatitis: A Study of More Than 115000 Individuals from the General Population. Clin. Chem..

[14] Eguchi K., Nagai R. (2017). Islet Inflammation in Type 2 Diabetes and Physiology. J. Clin. Investig..

[15] McGillicuddy F.C., de la Llera Moya M., Hinkle C.C., Joshi M.R., Chiquoine E.H., Billheimer J.T., Rothblat G.H., Reilly M.P. (2009). Inflammation Impairs Reverse Cholesterol Transport In Vivo. Circulation.

[16] Hage F.G., Szalai A.J. (2007). C-Reactive Protein Gene Polymorphisms, C-Reactive Protein Blood Levels, and Cardiovascular Disease Risk. J. Am. Coll. Cardiol..

[17] Ford E.S. (1999). Body Mass Index, Diabetes, and C-Reactive Protein among U.S. Adults. Diabetes Care.

[18] Michos E.D., Blumenthal R.S. (2009). Prevalence of Low Low-Density Lipoprotein Cholesterol With Elevated High Sensitivity C-Reactive Protein in the U.S. J. Am. Coll. Cardiol..

[19] Li C., Zhang Z., Luo X., Xiao Y., Tu T., Liu C., Liu Q., Wang C., Dai Y., Zhang Z. (2025). The Triglyceride–Glucose Index and Its Obesity-Related Derivatives as Predictors of All-Cause and Cardiovascular Mortality in Hypertensive Patients: Insights from NHANES Data with Machine Learning Analysis. Cardiovasc. Diabetol..

[20] Ma R., Cui L., Cai J., Yang N., Wang Y., Chen Q., Chen W., Peng C., Qin H., Ding Y. (2024). Association between Systemic Immune Inflammation Index, Systemic Inflammation Response Index and Adult Psoriasis: Evidence from NHANES. Front. Immunol..

[21] Guo W., Song Y., Sun Y., Du H., Cai Y., You Q., Fu H., Shao L. (2022). Systemic Immune-Inflammation Index Is Associated with Diabetic Kidney Disease in Type 2 Diabetes Mellitus Patients: Evidence from NHANES 2011-2018. Front. Endocrinol..

[22] Barter P., Gotto A.M., LaRosa J.C., Maroni J., Szarek M., Grundy S.M., Kastelein J.J.P., Bittner V., Fruchart J.-C. (2007). HDL Cholesterol, Very Low Levels of LDL Cholesterol, and Cardiovascular Events. N. Engl. J. Med..

[23] Tsvetkova V., Todorova M., Atanasova M., Gencheva I., Todorova K. (2025). Comparative Characteristics of the Immunometabolic Profile of Individuals with Newly Developed Metabolic Disorders and Classic Metabolic Syndrome. COVID.

[24] Sun H., Yang J., Ma L., Wu Y. (2025). Association between Hs-CRP/HDL-C Ratio and Risk of Prediabetes or Diabetes: A Cross-Sectional Study Based on NHANES 2015–2023. BMC Endocr. Disord..

[25] Finocchiaro S., Mazzone P.M., Ammirabile N., Bordonaro C., Cusmano C., Cutore L., Di Leo G., Faro D.C., Giacoppo D., Greco A. (2025). Anti-Inflammatory Pharmacotherapy in Patients with Cardiovascular Disease. Eur. Heart J. Cardiovasc. Pharmacother..

[26] World Health Organization (2000). Obesity: Preventing and Managing the Global Epidemic.

[27] Taskinen M.-R. (2003). Diabetic Dyslipidaemia: From Basic Research to Clinical Practice. Diabetologia.

[28] Xue K., Sun M., Zong C., Xing S., Xue H. (2025). Non-Linear Association of a Novel Inflammation-Lipid Composite Marker CRP/HDL with Insulin Resistance and Type 2 Diabetes: Findings from a Comprehensive National Cross-Sectional Study. Diabetol. Metab. Syndr..

[29] van Heck J.I.P., Ajie M., Joosten L.A.B., Tack C.J., Stienstra R. (2025). Circulating Inflammatory Proteins Are Elevated in Type 1 and Type 2 Diabetes and Associated to Complications. Diabetes Obes. Metab..

[30] Han F., Guo H., Zhang H., Zheng Y. (2025). Hs-CRP/HDL-C Can Predict the Risk of All Cause Mortality in Cardiovascular-Kidney-Metabolic Syndrome Stage 1-4 Patients. Front. Endocrinol..

[31] Gao Y., Wang M., Wang R., Jiang J., Hu Y., Wang W., Wang Y., Li H. (2024). The Predictive Value of the Hs-CRP/HDL-C Ratio, an Inflammation-Lipid Composite Marker, for Cardiovascular Disease in Middle-Aged and Elderly People: Evidence from a Large National Cohort Study. Lipids Health Dis..

[32] Ellulu M.S., Samouda H. (2022). Clinical and Biological Risk Factors Associated with Inflammation in Patients with Type 2 Diabetes Mellitus. BMC Endocr. Disord..

[33] Wu H., Ballantyne C.M. (2020). Metabolic Inflammation and Insulin Resistance in Obesity. Circ. Res..

[34] Berbudi A., Khairani S., Tjahjadi A. (2025). Interplay Between Insulin Resistance and Immune Dysregulation in Type 2 Diabetes Mellitus: Implications for Therapeutic Interventions. Immunotargets Ther..

[35] Liu M., Chen R., Zheng Z., Xu S., Hou C., Ding Y., Zhang M., Bao M., He B., Li S. (2025). Mechanisms of Inflammatory Microenvironment Formation in Cardiometabolic Diseases: Molecular and Cellular Perspectives. Front. Cardiovasc. Med..

[36] Varra F.-N., Varras M., Varra V.-K., Theodosis-Nobelos P. (2024). Molecular and Pathophysiological Relationship between Obesity and Chronic Inflammation in the Manifestation of Metabolic Dysfunctions and Their Inflammation-mediating Treatment Options (Review). Mol. Med. Rep..

[37] Casula M., Colpani O., Xie S., Catapano A.L., Baragetti A. (2021). HDL in Atherosclerotic Cardiovascular Disease: In Search of a Role. Cells.

